# Improving epidemiological projections for infectious diseases in Ghana: addressing methodological challenges

**DOI:** 10.1186/s41256-025-00449-3

**Published:** 2025-09-15

**Authors:** Verena Struckmann, Vincent Findeiss, Philip El-Duah, Jonathan Mawutor Gmanyami, Andrzej Jarynowski, Rexford Mawunyo Dumevi, Johanna Wildemann, Daniel Opoku, Vitaly Belik, Michael Owusu, Wilm Quentin, Christian Drosten, Johanna Hanefeld, John Amuasi, Reinhard Busse, Hanna-Tina Fischer

**Affiliations:** 1 Department of Health Care Management, Technical University of Berlin, Berlin, Germany; 2 German West-African Centre for Global Health and Pandemic Prevention, Berlin, Germany; 3 Institute of Virology, Charite, Universitätsmedizin Berlin, Berlin, Germany; 4 School of Public Health, Kwame Nkrumah University of Science and Technology, Kumasi, Ghana; 5 German West-African Centre for Global Health and Pandemic Prevention, Kumasi, Ghana; 6 Department of Veterinary Medicine, Freie Universität Berlin, Berlin, Germany; 7 Kumasi Centre for Collaborative Research in Tropical Medicine, Kwame Nkrumah University of Science and Technology, Kumasi, Ghana; 8 Chair of Planetary & Public Health, Universität Bayreuth, Bayreuth, Germany; 9 Robert Koch Institute, Berlin, Germany; 10 Bernhard Nocht Institute of Tropical Medicine, Hamburg, Germany; 11 Charité Center for Global Health, Berlin, Germany

## Abstract

The COVID-19 pandemic highlighted the essential role of disease modeling in shaping public health responses. However, models designed in high-resource settings often fail to capture disease dynamics accurately in lower-resource contexts like Ghana, where socio-ecological factors, infrastructure constraints, and data fragmentation complicate accurate predictions. In this Commentary, we examine the challenges of adapting global modeling approaches to Ghana’s context and propose strategies to improve their accuracy, relevance, and policy utility. These challenges were further compounded during the pandemic recovery period, when Ghana simultaneously faced outbreaks of Marburg virus and Mpox. These additional pressures—against a backdrop of rapid urbanization, increased human-wildlife interaction, shifting transmission dynamics, and environmental degradation—underscore the limitations of current modeling approaches. A key limitation lies in the difficulty of collecting raw, disaggregated data, accounting for sociocultural determinants, and capturing the complex interplay between disease dynamics and adaptive behaviors. Addressing these challenges requires valid, timely, and disaggregated data on social and epidemiological dynamics for model parameterization and validation. To examine the challenges faced in adapting global models for local use, we focus on Ghana’s unique context and argue for a rethinking of modeling approaches in this commentary. To mitigate potential harm, it is imperative to emphasize context-specific data, interdisciplinary input, and integration of social and economic factors, as foundational principles for future frameworks that can better support pandemic preparedness in Ghana and similar settings.

## Background

The COVID-19 pandemic underscored the value of disease modeling in epidemic response.^1^ However, models developed in high-resource settings face challenges when applied to low- and middle income countries (LMIC), particularly in Sub-Sahara Africa (SSA), like in Ghana.^2^ Ghana represents a particularly instructive case due to its high burden of some infectious diseases, active regional role in disease surveillance, and strong political commitment to digital health system strengthening. Ongoing efforts to expand real-time surveillance and integrate fragmented health data systems make it a valuable context in which to examine how global modelling approaches must be adapted—or rethought—for local use. Yet, critical variables for these models—such as disease-specific data on infection prevalence and immunity; population-specific parameters such as age structure, urban–rural distribution, mobility, and socio-economic diversity—are often incomplete or inconsistently captured.^3^ As a result, models developed during the pandemic often failed to accurately depict the rate of transmission in African countries, missing key socio-ecological factors influencing disease severity and progression. This Commentary discusses the unique epidemic modelling challenges Ghana faced during the COVID-19 pandemic and highlights core elements that need to be explored.

### Modelling challenges in Ghana

Ghana’s unique socio-ecological context reveals critical gaps between existing modeling frameworks and local epidemic realities. Ghana’s population is characterized by rich ethnic and linguistic diversity, with over 70 groups shaping a complex socio-cultural landscape that influences public health responses. About 30% of the population engages in agriculture and subsistence farming, while pastoralism can be mainly found in the north. Significant cross-border movement of people, goods, and livestock occurs along all borders with Ghana, complicating public health and biosecurity management. The pluralistic society, marked by regional practices, diverse religions, and community governance, shapes health behaviors, risk perceptions, and trust in institutions. Urban–rural disparities further hinder uniform public health efforts, with varying healthcare access, literacy, and communication modes. Trust in informal networks, elders, religious leaders, and traditional healers strongly shapes public opinion, especially in rural areas.^4^ This diversity underscores the need for culturally informed, context-specific disease modeling that can reflect how social norms and practices affect epidemic dynamics, non-pharmaceutical intervention (NPI) compliance, and health service uptake.

### Ghana’s COVID-19 data challenges

**Fig. 1:**
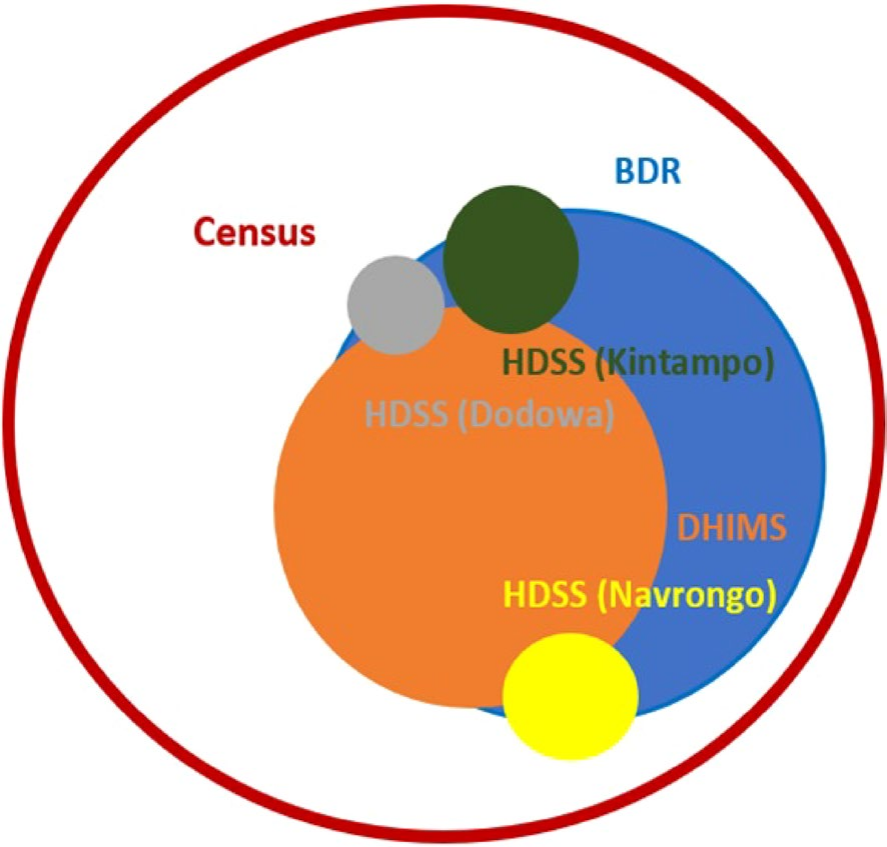
Scaled-area visualization of all-cause mortality in Ghana (2020–2021): reporting coverage across DHIMS2, births and deaths registry, HDSS sites, and the 2021 census

As of 7 April 2024, Ghana reported 172,075 confirmed COVID-19 cases and 1,462 confirmed deaths. However, underreporting issues suggest the actual deaths may be 27·75 times higher.^5^ Mortality data in Ghana is captured by multiple digital platforms including the District Health Information Management System (DHIMS), Lightwave Health Information Management System (LHIMS), Births and Deaths registry (BDR), the Health and Demographic Surveillance System (HDSS) sites, and individual mortuaries. During the COVID-19 pandemic, Ghana adopted additional digital surveillance tools, including the ‘Surveillance Outbreak Response Management and Analysis System’ (SORMAS), which supports the timely production of daily COVID-19 situational updates. Although these platforms complement each other, their differences in reporting mechanisms, data collection methods, and coverage areas create discrepancies and data duplication in national reporting^6^. Despite proactive testing efforts and initiatives to enhance diagnostic, reporting and surveillance capacities, data fragmentation, accurateness of data, limited diagnostic capacity, and underreporting remain significant barriers. These challenges undermine the reliability of infectious disease models, weakening their ability to predict transmission accurately, especially when using models based on higher-quality data from other regions. Addressing these issues will require a commitment to data integration and standardization across platforms, establishing real-time reporting mechanisms, and ensuring comprehensive data collection across the country (Fig. 1).

Circle areas are scaled proportionally to the number of deaths each source recorded, relative to the 2021 census estimate of 132,199 deaths (June 2020–June 2021), used as the reference for total mortality. For instance, BDR captured ~ 41.9% and DHIMS2 ~ 32.9% of this total. Circle radii were computed as the square root of the proportion to preserve area-based scaling. The outermost red circle represents the census (complete reference). Inner circles represent BDR, DHIMS2, and HDSS sites (Navrongo, Dodowa, Kintampo). Overlaps reflect areas where multiple sources captured the same events, based on field triangulation. Figure 1 illustrates substantial underreporting in routine systems and the need for integrated data to estimate true mortality. Since the majority of deaths go unrecorded, available sources like hospital records, BDR’s, and site surveillance were triangulated to estimate mortality rates. Node size reflects the proportion of true mortality captured during the COVID-19 pandemic, with overlap based on field studies and system integration.

The Ghanaian context reveals three particular methodological challenges in modeling that hinder accurate projections: adaptive NPI evaluation, robust data infrastructure, and social-behavioral parameterization:

- *Adaptive NPI Evaluation* NPIs such as mask mandates and social distancing need to be evaluated in terms of their effectiveness within local cultural contexts and with respect to community compliance. Developing adaptable, interdisciplinary metrics to assess NPI effectiveness in Ghana’s socio-cultural context is crucial. Unlike many higher-income countries (HIC), where NPI policy implementation tends to be more standardized and enforcement mechanisms are stronger, Ghana—like other LMICs—faces greater variability in compliance due to factors such as informal economies, communal living practices, and varying levels of trust in public health institutions. This makes context-sensitive evaluation frameworks essential for accurately capturing the effectiveness of NPIs.
- *Robust Data Infrastructure* Accurate projections depend on high-quality, real-time data. Expanding data triangulation from sentinel surveillance networks, improving model calibration to local contexts and integrating multiple data sources could improve accuracy and counteract issues of underreporting. Timely, complete, and integrated data flows across regions are essential for accurate disease modeling. While HICs often benefit from digitized health records and centralized data systems, Ghana and many LMICs still contend with fragmented, paper-based reporting systems and regional disparities in data coverage, leading to significant lags and data gaps in epidemic response.
- *Social-Behavioral Parameterization* Social behaviors and compliance with health guidelines vary significantly within Ghana’s diverse population. Incorporating public compliance levels, risk perceptions of community members, and demographic factors into the model can refine predictions and make them more reliable for real-time decision-making. This challenge is shared with other LMICs, where ethnic, linguistic, and religious diversity – as well as varying trust in authorities – complicates the use of standard behavioural assumptions. In contrast, many HICs benefit from more homogenous behavioral datasets and stronger institutional capacity for regular behavioral surveillance, allowing for more consistent incorporation of these parameters into models.

The effectiveness of each NPI can vary widely across regions due to differences in community compliance, which is influenced by public trust in health authorities, risk perception, socio-economic conditions and concurrent NPIs. This makes isolating the effect of individual interventions difficult.

Studies assessing NPIs in Ghana have mostly focused on selected areas or populations, creating an incomplete picture of nationwide compliance and effectiveness. Additionally, the lack of standardized evaluation frameworks complicates comparisons and makes it difficult to apply findings consistently across different communities.^7^ Addressing these challenges requires analyzing data from multiple populations and quantifying the differences between them to provide a nationwide picture. Culturally adapted metrics and regular community-based assessments to gauge public response, which would provide a more comprehensive understanding of the effectiveness of NPIs and the trust relationship between the public and health authorities, would also inform the development of targeted interventions.

### Modelling constraints from health systems data

COVID-19 case numbers and mortality rates in Ghana were inconsistently captured across platforms. While DHIMS provides nationwide data, it varies in accuracy and completeness due to infrastructure discrepancies at facility level and resource constraints.^8^ Further, Ghana’s National Health Insurance Management System omits data from those who rely on out-of-pocket healthcare or private health insurance. These discrepancies result in data gaps, making it impossible to analyze the effect of coverage (versus non-coverage), with only 54% of the population having an active National Health Insurance Scheme membership in 2021 and ultimately hinder the validation of predictive models that rely on accurate utilization and coverage data. Ghana’s testing capacity was also strained, and limited diagnostic availability reduced the ability to monitor true case numbers and variant spread effectively.

Due to the variety of healthcare providers and reliance on multiple funding mechanisms, data on healthcare utilization is fragmented across health services, including private providers. This impacts the predictive accuracy of models, particularly in estimating healthcare demand during a pandemic. Additionally, data discrepancies between DHIMS and HDSS, which differ in their data collection and reporting methods as well as in population coverage, make integration challenging. Ghana’s mortality data is also inconsistent: for example, in 2020, DHIMS reported 38,429 deaths, while the BDR reported 51,026; in 2021, DHIMS recorded 43,569 deaths, compared to 55,349 in BDR. Meanwhile, the 2021 census (covering June 2020–June 2021) reported 132,199 deaths^9^, indicating that DHIMS and BDR missed over half of all deaths. Improving model accuracy requires expanding sentinel surveillance, integrating local epidemiological patterns, and refining models to capture regional disparities.

COVID-19 diagnostics in Ghana relied heavily on polymerase chain reaction (PCR), requiring samples from the acute infection phase. Retrospective infection dynamics analyses are therefore only possible with archived samples collected during peaks of COVID-19 infections.^10^ However, due to limited long-term storage capacity and multiple laboratories involved, it is challenging to get access to these samples, if available. In the absence of PCR data, disease burden could also be retrospectively determined through serological data. However, obtaining this data can also be hampered by specific time windows within which evidence of past exposure can still be detected and cross-reactivity in assays due to co-circulating endemic human coronaviruses. The additional confirmatory tests needed to validate serological data also adds further financial and human resource costs that make it even more challenging to obtain. These diagnostic limitations constrain retrospective modeling efforts and reduce confidence in reconstructed transmission estimates.

## Towards more context-sensitive modeling frameworks

The following strategies aim to align modeling frameworks with Ghana’s social, data, and health system realities, increasing their validity and policy utility. Incorporating insights from epidemiology, virology, health system research and social sciences enhances model accuracy. Political scientists can help assess public risk perception and intervention compliance, virologists provide localized pathogen data, and health systems researchers identify access disparities. By integrating these perspectives, models can more effectively track infections while aligning with local behaviors and health system realities. However, investments in methodology should be carefully weighed against potentially more critical healthcare needs to ensure resource allocation remains responsive to Ghana’s broader public health priorities. To address Ghana’s modeling challenges, we propose targeted strategies:

1. Develop culturally adapted NPI metrics and evaluation
  Localized compliance metrics: Create metrics that reflect local behavioral patterns and conduct regular community surveys to capture public attitudes and local needs. These insights can help refine model parameters, providing a more accurate assessment of NPI impact.

2. Improve data infrastructure
  Integrate and centralize health data: Establish a digital repository that consolidates data from DHIMS, LHIMS, and HDSS for a unified, comprehensive source – acknowledging the need for phased implementation and capacity support. Expand sentinel surveillance sites across diverse regions to capture localized variations in transmission and improve data accurateness and completeness.
  Implement real-time reporting: Develop real-time data systems to support timely updates on mortality, and NPI compliance, enabling improved monitoring and faster, data-driven responses.
  Establish national reference biobanks: Centralize storage of biological samples to allow for nation-wide and retrospective studies, recognizing that such investments require sustained financing and technical capacity. Thus, these investments should be supported by cost-effectiveness evaluations that consider implementation and contextual constraints, such as financing, infrastructure, and workforce capacity.

3. Encourage socio-behavioral parameterization
  Engage local stakeholders: Build interdisciplinary teams of epidemiologists, social scientists, health system researchers, and community leaders to engage in participatory research and policy co-design to ensure models reflect local realities and demands. Collaboration improves relevance, trust, and adherence.

4. Establish guidelines and partnerships for local model adaptation
  Pilot testing and validation: Develop guidelines for local adaptation of global models, and test these in selected regions to document impact before scaling. Regional partnerships and international collaborations could provide technical and resource support to enhance model robustness. Knowledge transfer can be supported through training curricula, courses and technology transfer programs.

5. Strengthen a sustainable, data-driven and needs-oriented financing strategy
  Increase domestic health funding: Rebuild public trust and strengthen financing mechanisms to effectively pool public and private resources. Promote Program-Based Budgeting (PBB) to align spending with national priorities, invest in health infrastructure to support local research institutions, using enhanced data analytics for evidence-based and need-oriented budgeting.

## Conclusions

The challenges highlighted underscore the importance of a comprehensive, interdisciplinary, and context-sensitive approach to epidemic modeling in Ghana. Aligning models with Ghana’s health and social context, enables better data-driven policymaking. By investing in these foundational improvements, Ghana can build a resilient public health system, better protecting its population against future outbreaks. This approach would not only strengthen Ghana’s public health infrastructure but also set a valuable precedent for improving equity and applicability of epidemic modeling frameworks in other LMICs.

## Data availability 

Not applicable.

## Abbreviations

BDR: Births and deaths registry
COVID-19: Coronavirus disease 2019
DHIMS: District health information management system
HDSS: The health and demographic surveillance system
HIC: Higher income country
LHIMS: Lightwave health information management system
LMICs: Low- and middle income countries
NPI: Non-pharmaceutical intervention
PCR: Polymerase chain reaction
PBB: Program-Based Budgeting
SORMAS: Surveillance outbreak response management and analysis system
SSA: Sub-Sahara Africa
